# Developmental Trajectories of Hand Movements in Typical Infants and Those at Risk of Developmental Disorders: An Observational Study of Kinematics during the First Year of Life

**DOI:** 10.3389/fpsyg.2018.00083

**Published:** 2018-02-19

**Authors:** Lisa Ouss, Marie-Thérèse Le Normand, Kevin Bailly, Marluce Leitgel Gille, Christelle Gosme, Roberta Simas, Julia Wenke, Xavier Jeudon, Stéphanie Thepot, Telma Da Silva, Xavier Clady, Edith Thoueille, Mohammad Afshar, Bernard Golse, Mariana Guergova-Kuras

**Affiliations:** ^1^Department of Child and Adolescent Psychiatry, Necker-Enfants-Malades Hospital, APHP Assistance Publique-Hopitaux De Paris, Université Paris Descartes, Université Sorbonne Paris Cité, Paris, France; ^2^UMR 1129 Infantile Epilepsies and Brain Plasticity, Institut National de la Santé et de la Recherche Médicale, CEA, Université Paris Descartes, Paris, France; ^3^Institut National de la Santé et de la Recherche Médicale & Laboratoire de Psychopathologie et Processus de Santé, Université Paris Descartes, Université Sorbonne Paris Cité, Paris, France; ^4^Institut des Systèmes Intelligents et de Robotique, Centre National de la Recherche Scientifique UMR 7222, Université Pierre et Marie Curie Univ Paris 06, Sorbonne Universités, Paris, France; ^5^EA 3522, CRPMS, ED 450 Recherches en Psychanalyse et Psychopathologie, Université Paris Diderot, Université Sorbonne Paris Cité, Paris, France; ^6^Cellule Vidéo de l'Hôpital Necker Enfants Malades, Association A l'Aube de la Vie, Paris, France; ^7^Ariana Pharmaceuticals, Paris, France; ^8^Department of Visual Information, Vision Institute, Centre National de la Recherche Scientifique UMR S968, Institut National de la Santé et de la Recherche Médicale UMRS 7210, Université Pierre et Marie Curie Univ Paris 06, Sorbonne Universités, Paris, France; ^9^SAPPH, Fondation Hospitalière Sainte Marie, Paris, France

**Keywords:** infant at risk, hand movement, west syndrome, preterm, visually impaired mother, orality disorder, early hospitalization, developmental trajectories

## Abstract

**Highlights**

- The kinematics of hand movements (spatial use, curvature, acceleration, and velocity) of infants with their mothers in an interactive setting
  - are significantly associated with age in cohorts of typical and at-risk infants
  - differ significantly at 5–6 months of age, depending on the context: relating either with an object or a person.
- Environmental and developmental factors shape the developmental trajectories of hand movements in different cohorts: environment for infants with VIMs; stage of development for premature infants and those with West syndrome; and both factors for infants with orality disorders.
- The curvature of hand movements specifically reflects atypical development in infants with West syndrome when developmental age is considered.

We aimed to discriminate between typical and atypical developmental trajectory patterns of at-risk infants in an interactive setting in this observational and longitudinal study, with the assumption that hand movements (HM) reflect preverbal communication and its disorders. We examined the developmental trajectories of HM in five cohorts of at-risk infants and one control cohort, followed from ages 2 to 10 months: 25 West syndrome (WS), 13 preterm birth (PB), 16 orality disorder (OD), 14 with visually impaired mothers (VIM), 7 early hospitalization (EH), and 19 typically developing infants (TD). Video-recorded data were collected in three different structured interactive contexts. Descriptors of the hand motion were used to examine the extent to which HM were associated with age and cohort. We obtained four principal results: (i) the kinematics of HM (spatial use, curvature, acceleration, and velocity) were significantly associated with age in all cohorts; (ii) HM significantly differed at 5–6 months of age in TD infants, depending on the context; (iii) environmental and developmental factors shaped the developmental trajectories of HM in different cohorts: environment for VIM, development for PB and WS, and both factors for OD and; (iv) the curvatures of HM showed atypical development in WS infants when developmental age was considered. These findings support the importance of using kinematics of HM to identify very early developmental disorders in an interactive context and would allow early prevention and intervention for at-risk infants.

## Introduction

Early prevention and intervention are crucial in child psychopathology. Infancy is a period of rapid neurodevelopment and probably the best period for intervention, as it is a period of high neural plasticity (Benasich et al., 2014; Ismail et al., 2017). Many researchers have attempted to identify specific and reliable indicators of neurodevelopmental disorders, especially in social communication, in high-risk populations during the first year of life to recommend early interventions (Rogers et al., 2014). However, early clinical signs often lack specificity. One of the principal aims in studying neurodevelopmental disorders is to improve the early identification of such signs to propose targeted interventions, as early as possible, to improve social communication and clinical outcome. However, during the first year of life, social signals and any alterations are very difficult to identify (Zwaigenbaum et al., 2013).

HM appear to be a relevant cue for identifying early social signals. There are several reasons for assuming that social communicative gestures have evolved from HM (Gentilucci and Corballis, 2006). Montgomery et al. (2007) stated that humans not only produce HM to manipulate objects, but also to convey socially relevant information. Iverson (2010) argued that “motor acquisitions provide infants with an opportunity to practice skills relevant to language acquisition before they are needed for that purpose; and that the emergence of new motor skills changes infants' experience with objects and people in ways that are relevant for both general communicative development and the acquisition of language.” Thus, HM reflect key developmental milestones for the acquisition of communicative skills and the study of HM can provide relevant cues for early detection of developmental disorders.

### Characteristics of hand movements and their kinematics in typical infants during their first 10 months of life

Movements in infants have age-specific characteristics. Fetal/preterm general movements (GMs) show large variations (Prechtl, 1990) until 36–38 weeks gestational age (GA). Writhing movements are then observed between 36 weeks GA and 2 months post term. These movements are proximal, characterized by a small to moderate amplitude, and of slow to moderate speed (Prechtl et al., 1997). Fidgety movements appear between 2 and 5 months of age: they are more distal, of smaller amplitude and lower speed, and more variable in acceleration. In addition to GMs, infants exhibit various other spontaneous movements that increase in number and variety with age (Prechtl et al., 1997). At 3 months of age, voluntary, goal-directed, functional movements progressively replace spontaneous movements. These developmental changes in motor upper limb behaviors indicate changes in developmental stage: the infants' muscle strength increases to overcome the force of gravity and the use of environmental information induces an increase in varied, goal-oriented movements. These changes are associated with a shift from subcortical to cortical processing (Hitzert et al., 2015). Infants start to reach out at 3–5 months of age to explore objects and the environment with their hands (Thelen et al., 1996). By 9 months, infants begin to develop clear communicative gestures, such as pointing, which paves the way for language development (Iverson and Goldin-Meadow, 2005; Iverson, 2010).

Recent technical advances have provided opportunities to automatically detect, assess, and track clinical developmental features, such as HM in infants. Kinematics is referred to as the “geometry of motion” by describing the position, velocity, and acceleration/deceleration of points of the described object and movement units, of which the combination measures the trajectory, curvature or jerkiness, smoothness, straightness, complexity, and repertoire of movements (von Hofsten, 1991; Thelen et al., 1996; Berthier and Keen, 2006; Meinecke et al., 2006). Kinematics of GMs are characterized by smoothness, assessed by constant changes in the angular values (Karch et al., 2008) and high complexity, without regular periodicity (Meinecke et al., 2006). Little longitudinal data concerning kinematics of spontaneous HM have been collected on infants. Gima et al. (2011) showed that infants' spontaneous movement involves chaotic dynamic systems that are capable of generating voluntary skill movements around 3–4 months. Waldmeier et al. (2013) showed a decreasing trend of statistical persistence in the acceleration time series with maturation, from birth to 2–3 months of age. Zoia et al. (2013) have studied the kinematics of upper limb movements from fetal life to 12 months of age. They particularly found two results: the longer movement duration and deceleration time at 22 weeks of gestation for movements toward the eye rather than the mouth, re-emerge at 4 months of postnatal life. They also showed at 4 months a reorganization of the length of the deceleration phase of the movement according to environment differences. Their results possibly suggest reorganization phases according to the novel environment.

Kinematics have largely been used to study infant reaching and prehension (Berthier and Keen, 2006; Lynch et al., 2008; Gonçalves et al., 2013). A period of pre-reaching, specific to toy oriented gestures, has been described (Bhat and Galloway, 2006). The smoothness of the reaching movement improves from 2 to 7 months of age (Lee et al., 2011), but independently of straightness (Thelen et al., 1996). Konczak et al. (1995) described this second period of reaching as the “fine-tuning” of the sensorimotor system that begins at 6–7 months, with smoother movements, fewer movement units, and an increase in straightness. Although reaching parameters become more stable by 8 months (Thelen et al., 1996), the time period for reaching kinematics remains controversial. Infants reliably grasp for objects within their workspace 3 ± 4 months after the onset of reaching, stable patterns of temporal coordination across arm segments begin to emerge at 12–15 months of age and continue to develop up to the 3rd year (Konczak and Dichgans, 1997).

Marcroft et al. (2014) reviewed the advantages and limitations of various methods applied to assessing spontaneous GM in high-risk infants. Indirect (video cameras or 3D motion capture) or direct sensing (through sensors worn by the patient) both capture limb movements, but differ in their temporal and spatial resolution. The main advantage of video-based movement analysis is the recording of spatial data in addition to temporal measurements, in a natural setting. Sensor approaches have much higher temporal resolution, but are less feasible in everyday clinical practice and carry on artifact-related issues.

Even though video based analysis allows assessment of movements under natural conditions, infants are generally assessed lying or sitting, without any specific task. Very few studies have described the kinematics of HM during interactions with the caregiver, although these are the most relevant situations to assess genuine interactive processes (Avril et al., 2014; Leclère et al., 2016).

### Characteristics of hand movements and their kinematics in at-risk infants

The literature on the kinematics of HM mostly concerns preterm infants. The variation of these patterns (intrinsic movement features and their repertoire) has been used to predict later neurological outcome. Atypical motor development in preterm and full-term infants (limited variation, poor repertoire, fluency, and complexity), especially the presence of cramped synchronized GMs which are more stereotypical, predict later developmental impairment and cerebral palsy (Hadders-Algra, 2004; Einspieler et al., 2012). The poor repertoire of GM has shown its utility in predicting neurodevelopmental outcome at two (Beccaria et al., 2012) and two-and-a-half years of age (Kodric et al., 2010). In particular, poor quality of fidgety GM at 3–5 months has been reported to predict minor neurological dysfunction later, at school age (Hadders-Algra and Groothuis, 1999). However, earlier GMs, during the writhing period, are considered to be less reliable in predicting cerebral palsy (Prechtl et al., 1997).

The clinical assessment of hand movements in at-risk infants requires training and expert clinicians. Nonetheless, kinematics is an important tool to detect specific cues of HM, which can then be more precisely clinically assessed. Ohgi et al. (2008) found that HM in infants with brain injuries were more unstable and less predictable than the movements of low-risk infants in a longitudinal study of spontaneous movements of preterm infants. Adde et al. (2009) classified non-fidgety vs. fidgety movements in preterm infants using computer-based video analysis. Kinematics have shown superiority over clinical assessment to detect specific features of HM: clinical assessment correctly identified all infants with neurodevelopmental impairment, including cerebral palsy, by 3 months, but only the stereotypy score of limb movement, derived from kinematics, discriminated the appearance of cerebral palsy from other developmental impairments (Philippi et al., 2014).

Infants at risk of autism spectrum disorders (ASD) are particularly studied during their first year of life. The detection of early signs is the first step to intervene and improve the prognosis of neurodevelopmental disorders (Rogers et al., 2014). Although later communicative gestures in ASD children are well-known, relatively little attention has been given to specific motor and movement development as a potential diagnostic risk marker for ASD during the first year of life, until recently. Such studies are mostly retrospective (e.g., home movies) and lack systematic motor assessment (Zwaigenbaum et al., 2013). Only one assessed early movements by video analysis, but none used kinematics. Phagava et al. (2008) found a preponderance of writhing movements in ASD children (70% in ASD vs. 12.5% in controls), and the absence of or abnormal fidgety movements were found in 50% of ASD vs. 11% of control children. However, no statistical significance was reported due to the small sample size. Two other studies coded home videos using a standardized movement analysis system. Esposito et al. (2009) assessed 0 to 5-month-old infants in a supine position with “manual” frame-by-frame video rating of movements. Infants with ASD exhibited lower levels of both “static” and “dynamic symmetry” than both infants with intellectual disability and controls. Teitelbaum et al. (1998) assessed GMs in 4 to 6-month-old infants on home videos using the Eshkol–Wachman Movement Analysis System and found disturbances in the shape of the mouth and development milestones (lying, righting, sitting, crawling, and walking) in infants who later showed autistic features, but did not focus on HM. No specificities have yet been found for motor function nor HM.

### Study goals

This longitudinal and observational study aimed to assess HM in infants at risk for developmental disorders and controls, followed from the age of 2 to 10 months, during mother-infant interactions. We hypothesized that HM may reflect early specificities of gesture and communicative development, which are clinically difficult to assess. We selected five groups of at-risk infant populations: two populations for neurodevelopmental risk, including ASD (epileptic infants with West syndrome; premature infants); two for specific environmental factors [infants being hospitalized immediately after birth and infants with visually impaired mothers (VIMs)]; one for both risks (infants with orality disorders); and one control population. We examined the longitudinal development of HM under natural interactive conditions with the mother, during three communicative situations, to differentiate toy-directed and person-directed movements.

We explored the kinematics of HM using several kinematic descriptors to characterize the spatial use, curvature, acceleration, and velocity of HM asking the following research questions:

- Do HM differ with age in all cohorts?
- Do HM differ by context: person vs. object directed?
- Do HM differ by cohort: typical vs. at risk to be atypical?
- Can we use HM as clinical markers for neurodevelopmental disorders?

## Materials and methods

### Design and participants

At risk participants were recruited from the Necker Enfants-Malades Hospital, Paris, and healthy or TD infants from maternal and infant protection institutions, pediatric consultations, or by proxy, from February 2004 to March 2013. Six cohorts of infants with and without risk were selected (Table 1):

1. West Syndrome (WS): The WS cohort was particularly important because it leads to ASD in 7–25% of cases and to developmental delay or learning disorders in 70–90% of cases. Prior studies have already shown that early assessment of interactions is an early predictor of later developmental delay or ASD (Ouss et al., 2014).
2. Orality Disorders (OD): Infants with oral disturbances (early oral feeding disorder or metabolic disease that require a special diet: enteral feeding or strict diet). This cohort was chosen to test the hypothesis that an early dysfunction of orality, with a medical cause, could lead to later communicative, developmental, or interactive dysfunction.
3. Preterm Birth (PB): This cohort was chosen because it has been shown that PB is a risk factor for later neurodevelopmental disorders, including ASD (D'Onofrio et al., 2013).
4. Visually Impaired Mothers (VIM): Infants with VIMs. This cohort was chosen because the mothers' sensory deficit may influence the communication and development of the infant.
5. Early Hospitalization (EH): Full-term babies with early hospitalization during the first 3 months after birth for acute reasons. This cohort was selected to test the impact of a minor environmental effect during the first year of life.
6. Typical Development (TD): Infants with typical development were included as controls

**Table 1:** Cohort characteristics.

**Cohort**	***N***	**Description**	**Specific exclusion criteria**
1- West Syndrome (WS)	25	WS with following etiologies: 7 idiopatic 12 unknown origin 6 structural/metabolic origin or genetic disease: 2 pachygyria, 1 Tuberous Sclerosis Complex, 1 Rett syndrome, 1 neonatal brain ischemia, 1 prematurity (32 weeks) DQ: 12 ≥ 70, 13<70	None
2- Orality disorders (OD)	16	Pierre Robin syndrome; phenylketonuria; citrullinemy; arginosuccinic aciduria; deficit in ornithine carbamyl transferase (OTC); leucinose; methylmalonic acidemia; propionic acidemia; tyrosinemy, or glycogenosis DQ in the normal range	None
3- Preterm Birth (PB)	13	Infants born between 30 and 37 gestational weeks (mean 32.3 weeks) Mean hospitalization: 40.8 days Birth weight varied from 665 to 3100 g (mean 1,619 g) Seven were admitted to the neonate intensive care unit for 1–17 days (mean 8.5 days) Five babies were small for their gestational age DQ > 90	No neurological impairment at the first neurological examination
4- Visually impaired Mothers (VIM)	14	Six mothers with visual impairment Six blind mothers, of whom two were blind from birth, one who gradually become blind at 21 years of age, one during childhood, and two during adolescence; and two with pigmentary retinitis, who gradually lost their vision during the study	DQ < 85
5- Early Hospitalization (EH)	7	Hospitalization for persistent fever, partial respiratory failure, diarrhea, bronchiolitis. Hospitalizations occurred either immediately post-partum or during the first 3 months of life Recovery during hospitalization in <10 days	DQ < 85
6- Typical Development (TD)	19	Typical development	DQ < 85

Developmental Quotient (DQ) was measured by the Brunet-Lezine (BL) scale at 12 months, which estimates the developmental quotient in four domains: posture, coordination, language, and socialization, based on standardized developmental quotients available for 0- to 30-month-old French toddlers (Josse, 1997).

Exclusion criteria were as follows: parents' refusal to consent to follow-up assessment and/or to the research protocol, families who lived too far from the hospital, or families who could not complete the questionnaires due to an insufficient knowledge of French. This study was approved by the Institutional Review Board (Comité de Protection des Personnes from the Groupe-Hospitalier Necker Enfants Malades, IRB registration number 00001072), and parents gave written informed consent after receiving verbal and written information on the study. CNIL procedures (Commission Nationale Informatique et Libertes) for data anonimity processing were respected.

### Video recordings

**Figure 1:**
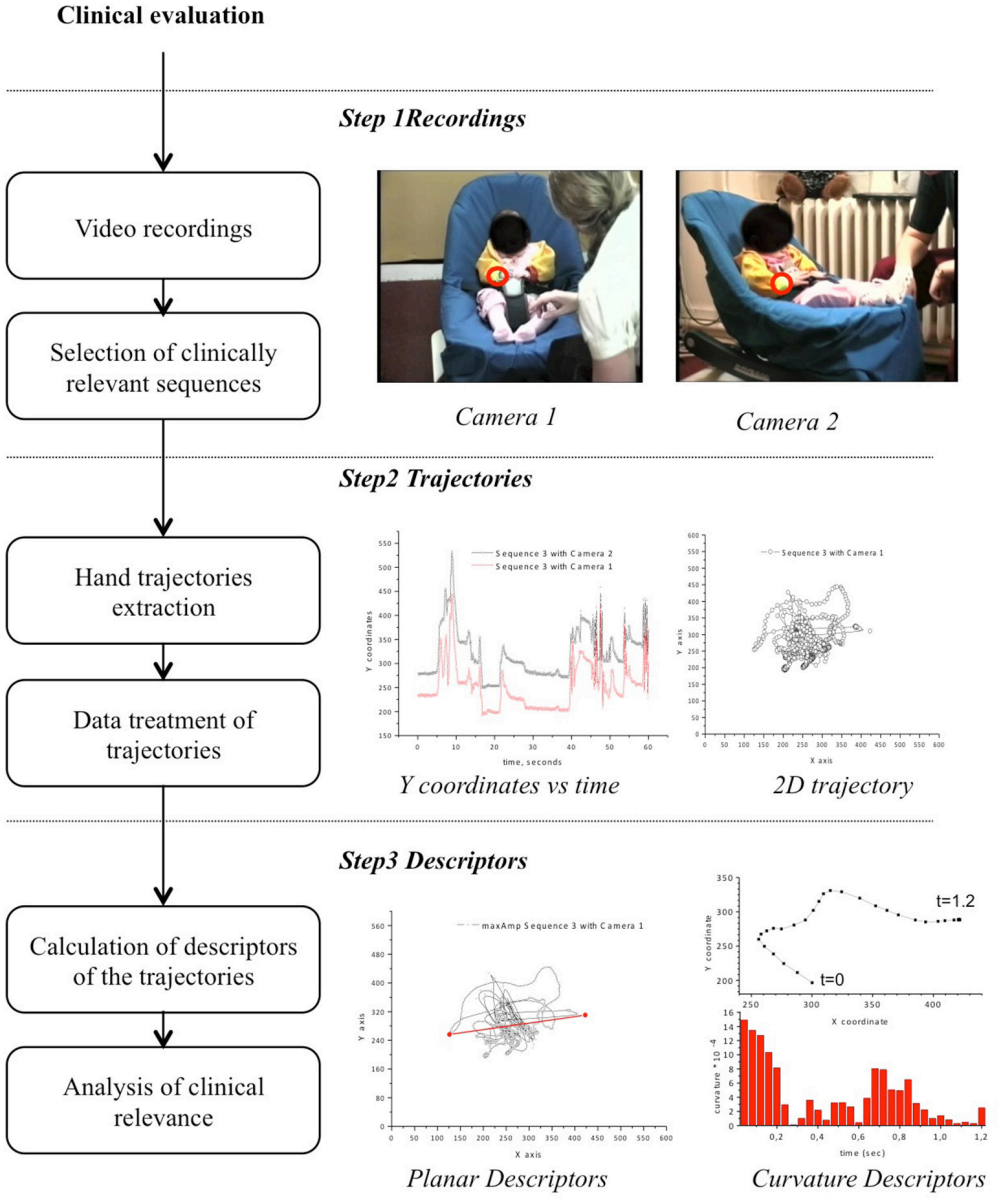
Experimental workflow and data processing. In Step 1, the position of the two cameras is illustrated with two frames from the recordings. The wristband, which is bright yellow, is shown in the red circle. The mother was positioned slightly higher in all the recordings, because of the position of the baby-chair on the floor and the mothers' seated position. The mothers' indicated position was on the left of the infant as shown on the picture, but exceptions were sometimes observed during the recordings. In Step 2, the 2D coordinates of the hand are extracted from each of the video recordings. The Y coordinates of the hand movements of a 9-month-old from the EH cohort, recorded with the two cameras during sequence 3, are shown in black and red (central panel). The Y coordinates are slightly shifted due to the positions of the cameras. The hand trajectory expressed as a line connecting the planar coordinates, recorded with camera 1 for the same recording as previously shown, is shown in the right panel. In Step 3, the descriptors of the hand movements are calculated from the trajectories. The maximum amplitude of the trajectory is shown in red (bottom central panel). The calculated curvature at each point of the trajectory is presented in the bottom right panels in which the first 1.2 s of the trajectory are plotted and the associated calculated curvatures at each point (and respective time, indicated on the axis) are presented as columns.

The infants' HM were assessed during standardized play sessions in interaction with the mother. They were recorded at various timepoints from the age of 2 to 10 months. Two synchronized cameras (face and profile, see Figure 1, top panel) recorded the movements in two dimensions while the infant was sitting in a baby-chair. The standardized situation was divided into three sequences: 3 min of free play in which the mother was instructed to interact “as usual” without any toy (sequence 1), 3 min of play with a cuddly giraffe (sequence 2), and 3 min of play with the mother singing a well-known nursery rhyme accompanied by rotating her own hands (sequence 3).

One minute was extracted from each 3-min video sequence (thus 3 min per infant per session) according to two criteria: the infant's hands should be visible for at least part of the sequence (e.g., the mother is not leaning toward the infant) and the minute was that with the greatest amount of interaction between the mother and the infant. This duration does not reflect the entire complexity of interactive behaviors, but in the field of social and clinical psychology, Ambady and Rosenthal (1992) have shown that predictions based on recorded observation of even under 30 s did not differ significantly from predictions based on 4–5 min observation.

### Extraction of hand trajectories

The coordinates of the hands were computed from the video images using a tracking framework implemented in a software that was designed and developed for this purpose (software and documentation available in Supplementary Material). The tracking framework comprised three steps: prediction, observation, and estimation. The prediction step estimated potential locations of the hand using a dynamic model. The observation step estimated the probability associated with the predicted location, using a computed likelihood measure, based on the data provided by the camera. Finally, an estimation of the state of the hand (here limited to its positions and velocities in the picture) was performed. A yellow wristband was used for the right hand for the observation step. The observation was based on the Bhattacharyya distance, which measures the likelihood between color histograms computed on a region-of-interest around the predicted location and on a hand-selected one in the first frame of the sequence (Czyz et al., 2005). For the other two steps, we developed an approach using a bootstrap-based particle filter with a first-order model as the HM were highly non-linear with abrupt changes in direction and speed (Isard and Blake, 1998; Hue, 2003).

The infants' hands were often occluded in the video, as the parent could interact freely with the infant. We used an approach combining tracking with detection (Czyz et al., 2005) to minimize this problem. This was performed by adding a Boolean variable to the state vector associated with each particle: its value was set to zero if the particle was in detection mode or to one if the particle was in the tracking mode. The transition from one mode to the other was managed via a transition matrix. In the tracking mode, the state of the particle was determined according to a classical particle filter-based algorithm. In the detection mode, the state of the particle was randomly determined using a computed probability map, based on the entire image.

### Data processing of hand trajectories

Each extracted trajectory consisted of 1,500 pairs of x and y coordinates (25 frames per second, generating 1,500 pairs of coordinates during the 60 s, see Figure 1, second panel). The frames in which the hand was not visible were clearly indicated in each trajectory as missing coordinates for these timepoints. Incomplete trajectories missing more than 30% of the coordinates, either due to occlusion of the camera by the mother or losing the target wristband by the tracking framework, were removed from further analysis. The trajectories obtained were normalized using a fixed reference system present in the settings of each video recording (Figure 1) to account for differences in the camera zoom parameters. Normalization was performed on all trajectories and 95% of the normalization factors ranged between 0.8 and 1.22, with a few outlier trajectories that required greater correction. Forty-one percent of the trajectories required less than a 5% correction. The recordings between the two cameras were synchronized and, in principle, should have allowed reconstruction of the trajectory in three dimensions. Such reconstruction was not possible because of the accumulation of missing data, asynchronously observed by the two cameras, for each trajectory. However, it was recently reported that 2D motion capture with appropriately defined movement descriptors can be a powerful method for detecting clinically relevant changes (Marcroft et al., 2014). We thus decided to perform a combined, independent analysis of the videos recorded by each camera as the two cameras provided orthogonal views of the infants' HM.

### Descriptors of the HM to explore hand trajectories

**Figure 2:**
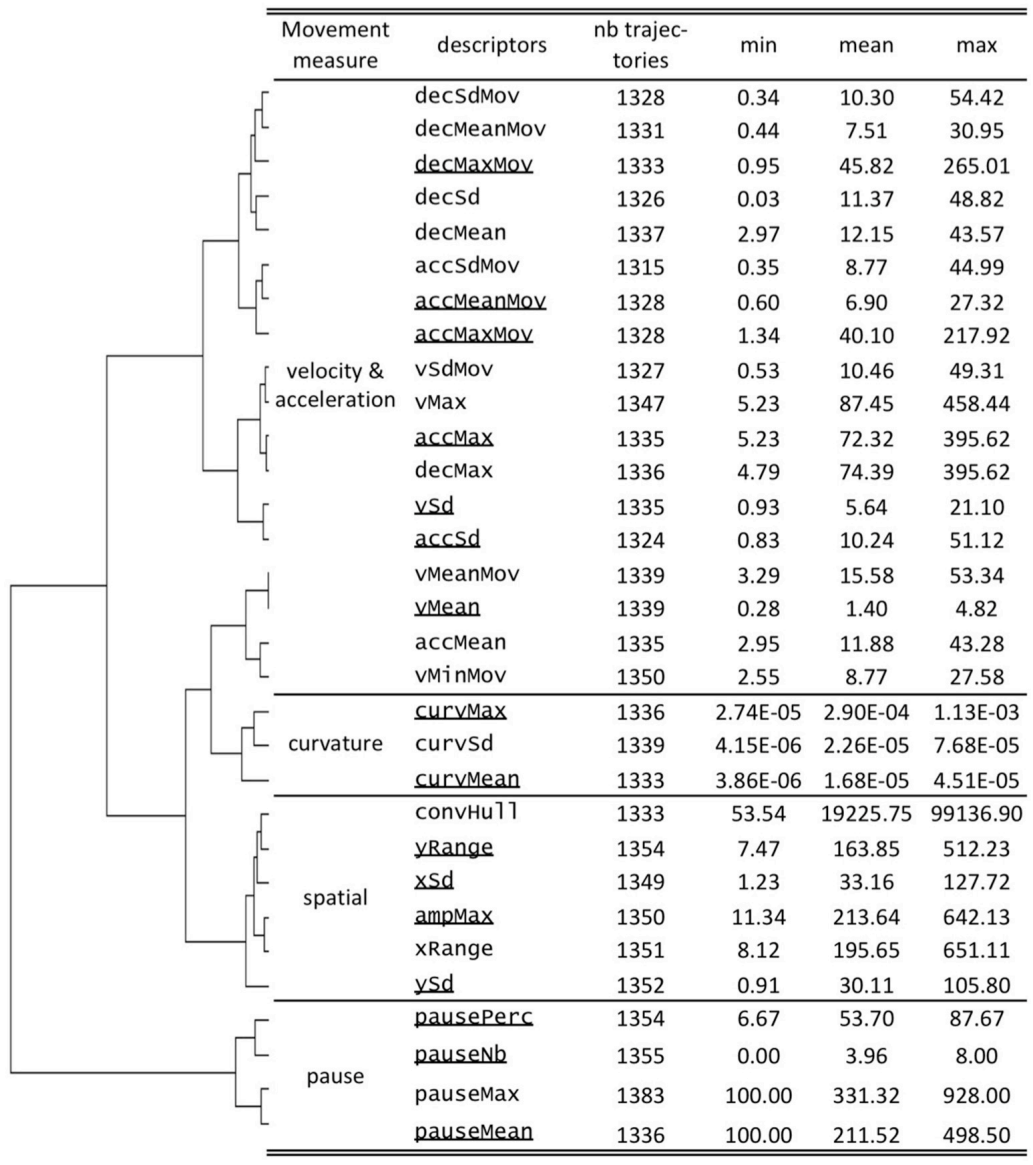
Calculated descriptors of the trajectories. Data from a hierarchical clustering, using the Ward minimum variance method of the descriptors, is presented as a dendogram to the left of the descriptors. Descriptors selected to be representative for each group of movement measures, based on their degree of dissimilarity, are underlined.

The coordinates of the trajectories were used to calculate descriptors characterizing the infant's HM. Four types of descriptors were calculated (Figure 2), covering the main descriptors already reported in the literature (Marcroft et al., 2014).

1. Velocity and acceleration: The six descriptors characterizing the velocity were calculated throughout the entire recorded sequence, taking into account the pauses (see below). Descriptors of velocity, when the hand was considered to be in motion, were calculated by removing the intervals considered to be pauses and are labeled as “Mov” to distinguish them from the descriptors of the overall sequence. The maximum achieved velocity (vMax) was considered from the entire sequence, whereas the minimum velocity was considered only during motion (vMinMov). The change in velocity was considered separately as positive (acceleration) and negative (deceleration) values both during the entire sequence and only during motion.
2. Curvature of the hand movements: A set of descriptors on the curvature of the trajectories was calculated, using a standard definition of the curvature (κ) of plane curves in Cartesian coordinates as γ(t) = (x(t),y(t))
  $$\kappa =\frac{\left|x\prime y\prime \prime -y\prime x\prime \prime \right|}{{\left(x\prime^{2}+y\prime^{2}\right)}^{3/2}}$$
  The curvatures observed throughout each trajectory were summarized as the mean (curvMean) and maximum (curvMax) values, as well as the variability expressed as curvSd (Figure 2).
3. Explored space: We calculated six descriptors, independently of the origin of the coordinate system, to describe the space explored by the hand: the maximum distance observed on the two axes (xRange, yRange) and the standard deviation (xSd, ySd). The two measures provide an estimate of the amplitude of the movements on each axis, with the standard deviation being less sensitive to outlier coordinates. We also calculated the maximum distance between any two points of the trajectory, using the FarthestPair java library (http://algs4.cs.princeton.edu/code/) with minor modifications. The planar surface explored by the hand during the recording session was approximated using the Convex Hull calculation implemented in the GrahamScan.java library (http://algs4.cs.princeton.edu/code/). Descriptors related to the exploration of the space are illustrated for one representative trajectory in the third panel of Figure 1.
4. Movement pauses: A final set of descriptors was defined to describe the pauses of the HM. We defined a pause as part of the trajectory in which the velocity was lower than a specific threshold. A minimum duration of 4 sec was defined as the period to be considered as a pause. Pause boundaries were located when two consecutive points with a velocity higher than the threshold were observed in the trajectory. The pauses were evaluated by their total number (pauseNb) for the duration of the sequence and their mean (pauseMean) and maximum (pauseMax) duration. A parameter that considered the relative time spent motionless by the infant during the sequence video recording was also calculated (pausePerc). When no pause was observed in a sequence (pauseNb = 0), the other pause parameters were set as missing values.

The outlier values for each descriptor were evaluated and used to remove either aberrant points in the trajectory or aberrant values of a descriptor for a specific trajectory. The threshold to consider a value as aberrant was set at the median plus eight times the standard deviation. The points of the trajectory or the calculated descriptor were then considered to be outliers and were replaced with missing values.

### Statistical analysis

The statistical analyses were performed on the calculated descriptors using the R statistical package (version 3.0.1). The similarity between different descriptors was assessed using hierarchical clustering based on the Pearson correlation index and only sixteen descriptors with correlation below 0.8 were retained. The normality of the distributions were evaluated using the Wilk–Shapiro test. None of the variables was considered as normally distributed and the differences between cohorts were thus assessed using the non-parametric Kruskal–Wallis one-way analysis of variance test based on the ranks. The test was followed by a Dunn test to more precisely determine the groups with different effects. The interactions between age and cohort and between age and sequence were tested with a type III ANOVA.

Infants with WS have a developmental age (DA) that is lower than the norm, which could bias the results for this specific cohort. We used the DA measured with the Brunet-Lezine scale to control for this effect.

## Results

### Video-based analysis allows good quality extraction of hand trajectories

**Figure 3:**
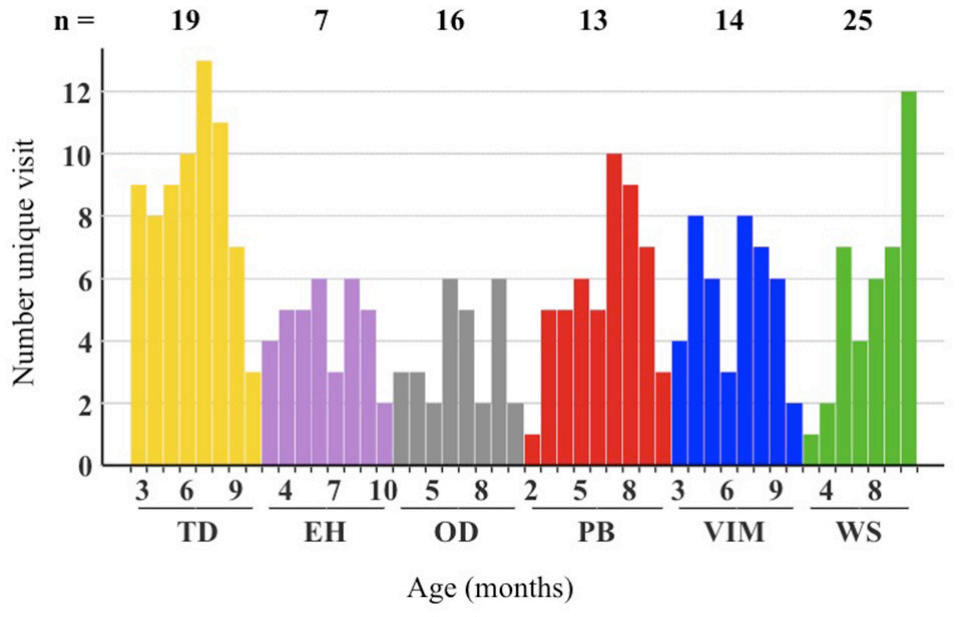
Number of visits per age of the various cohorts. The number of visits is shown for each month, separately for each cohort. The total number of infants in each cohort is indicated at the top. The x-axis (in months) is repeated for each cohort.

A total of 94 infants (47 girls and 47 boys) were included in the study. For all infants, recordings were performed at various time points from the age of 2 to 10 months, totaling 289 entries. The recruitment and follow-up of the infants during these 8 months was heterogeneous across cohorts (Figure 3) with a weighted average age similar for all cohorts (~6 months), except for the WS cohort, for which infants were recruited at a slightly higher age, thus shifting the weighted average age to 8 months. The follow-up of the WS cohort was concentrated in the last few months of the 2–10 month period, as the symptoms of WS often appear between 4 and 7 months of life. The average number of visits per infant also varied across cohorts with the highest number of visits for the EH cohort (5.3 visits per infant) and the lowest number for the WS cohort (1.6 visits per infant).

Each recording session consisted of three video sequences that were followed by two cameras, generating a total of 1,734 video sequences. The tracking framework generated 1,446 trajectories (83% of successful coordinate extractions). The main reasons for unsuccessful extraction of the coordinates were deviations from the protocol, either in the compliance of the observational setup (e.g., the mother remaining between the infant and the camera for the entire duration of the recording or a wristband of the same color as the infant's clothes). When possible, manual tracking of missing episodes of the target wristband was conducted (wristband not visible but the hand not obstructed by the mother) to extract the coordinates. The obtained trajectories were equally distributed between cameras and sequences. The comparison between the y coordinates of the two orthogonally positioned cameras (Figure 1, second panel) suggested that the trajectory extraction was reproducible. This was further confirmed by the lack of effect on the camera on all measures, based on the vertical coordinates (min *p*-value: 0.5), as well as the velocity, acceleration, and pauses for which the two cameras were expected to capture similar information. After removing trajectories containing <70% of the expected coordinates, a total of 1,355 trajectories was obtained and used to derive the descriptors of the hand trajectories (Table 2).

**Table 2:** Participant characteristics and follow-up.

	**Number of infants (% boys)**	**Average number of visits**	**Weighted average age (months)**	**Camera 1**			**Camera 2**			**Total selected sequences**
				**Seq 1**	**Seq 2**	**Seq 3**	**Seq 1**	**Seq 2**	**Seq 3**	
Control (Ctrl)	19 (63%)	3.9	6.2	67	64	63	66	56	62	378
Visually impaired mothers (VIM)	14 (50%)	3.1	6.3	39	36	36	36	34	33	214
Early hospitalization (EH)	7 (57%)	5.3	6.3	33	29	34	34	29	33	192
Orality disorders (OD)	16 (31%)	1.8	6.6	24	25	19	25	22	19	134
Preterm birth (PB)	13 (77%)	4.2	6.5	48	47	46	49	45	46	281
West syndrome (WS)	25 (36%)	1.6	8	30	27	23	28	23	25	156

*Camera 1 provided the front view; Camera 2 provided the side view of the infant (see Figure 1); Seq 1 is the free interaction; Seq 2 is the play with the cuddly giraffe; Seq 3 is the interaction when the mother is singing a nursery rhyme*.

### Kinematics of infants' HM in the three interactive settings

Descriptors were extracted from the trajectories to characterize four aspects of the infants' HM: (i) their velocity and acceleration to assess smoothness, which characterizes the development of reaching (Thelen et al., 1996); (ii) their shape through the curvature of the movements; (iii) the space explored through the amplitudes and their variability for each axis, as well as for the entire trajectory; and (iv) the overall activity during the recorded sequence (through different measures of the pauses) (Figure 2).

The HM in the three environmental settings were different for all descriptors, except for the number of pauses, for which the mean value of approximately four pauses per recorded sequence was observed overall. In all cases, the sequence in which the infant played with the cuddly giraffe was different from the other two when the sequence effect was significant. The infants' HM in this sequence were characterized by larger amplitudes, higher velocity and accelerations, shorter pauses, and more overall time spent in movement. Only three descriptors differed (xSd, ySd, and PausePerc) between the free play and nursery rhyme sequences. The amplitudes of the movements (xSd, ySd) and the time spent in movement (PausePerc) were slightly lower for the sequence with the nursery rhyme. The strong similarity of the movements in the latter two sequences probably reflects the similarity of the communication between the infant and the mother in these two settings, as opposed to playing with the cuddly giraffe.

### Kinematics of infants' HM over time

There was a significant age effect for all descriptors, except for those related to pauses (*p* = 0.28). Spatial, curvature, acceleration, and velocity descriptors were highly dependent on age, with the strongest associations for the spatial descriptors, more specifically vertical amplitude (Table 3). The 40% greater amplitude (yRange *p*-value of 6e-25) reflects, in part, the growth of the infants' arms during the study. The increase in movement velocity with age was also highly pronounced: from a 25% increase for vMean to a 50% increase for the acceleration metrics.

**Table 3:** Hand movement descriptors.

**Descriptor of HM**	**At 3 months**		**At 10 months**		**Age impact^¶^**	**Settings impact^¶^**	Age^*^Settings^¶¶^
	**Avg**	**StdDev**	**Avg**	**StdDev**			
decMaxMov	36.1	44.25	54.63	55.47	^***^	^***^	NS
accMeanMov	5.67	4.11	7.61	4.32	^***^	^***^	NS
accMaxMov	32.51	42.41	44	43.06	^***^	^***^	NS
accMax	57.26	71.18	89.68	84.51	^***^	^***^	NS
vSd	4.91	3.6	6.45	3.61	^***^	^***^	^*^
accSd	7.99	8.09	12.28	9.87	^***^	^***^	NS
vMean	1.25	0.79	1.56	0.83	^***^	^***^	^*^
curvMean	1.91E-05	7.18E-06	1.74E-05	7.13E-06	^***^	^**^	^**^
curvMax	2.97E-04	1.91E-04	3.01E-04	2.01E-04	^**^	^**^	NS
yRange	135.03	76.93	189.75	89.92	^***^	^***^	^**^
xSd	24.59	16.51	39.38	24.21	^***^	^***^	NS
ampMax	175.79	111.42	252.73	124.77	^***^	^***^	NS
ySd	24.2	14.1	34.8	20.1	^***^	^***^	^*^
pauseNb	4.07	1.38	4.13	1.36	NS	NS	NS
pauseMean	203.87	61.46	209.13	68.84	NS	^***^	^*^
pausePerc	53.76	16.1	55.12	14.38	NS	^***^	^*^

Avg, mean; StdDev, standard deviation;

¶ Kruskal–Wallis one-way ANOVA p-value;

¶¶ ANOVA type III, p-value of interaction;

*** p ≤ 10^−3^;

** p ≤ 10^−2^;

* *p ≤ 0.05; NS, non-significant*.

### Kinematics of infants' HM: time points and setting

The HM were also different by context (Table 3). We tested the relationship between the HM, the three experimental interactive settings, and the infants' age in the interaction between age and sequence in a linear regression model for the various descriptors. The interaction between age and sequence was significant (*p* < 0.05) for descriptors associated with vertical amplitude (ySd, yRange), velocity (vMean, vSd), and time spent in motion (pauseMean, pausePerc), but not acceleration, suggesting that the evolution of the movements differed depending on the situation. The cuddly giraffe sequence was found to be different from at least one other sequence.

**Figure 4:**
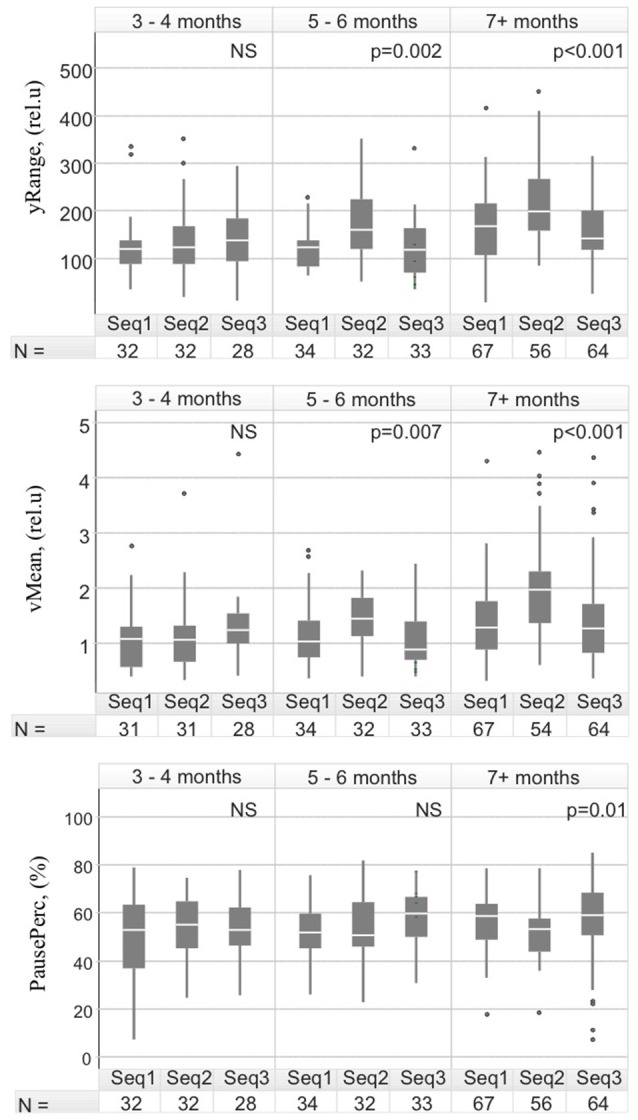
The age at which differences in hand movements appear in the control cohort. The differences in the descriptors related to the vertical range **(Top)**, mean velocity **(Middle)**, and time spent without movement during the 60 s of recordings **(Bottom)** are presented for three age spans: for 3- and 4-month-old infants; (TD infants did not have recordings at 2 months) for 5 and 6 months and for 7 months and later. The number of sequences used to calculate the statistics is shown in the tables below each plot. The median values are represented as white lines in the box-plots and the lines represent the 95th percentile of the data. Outliers are indicated as additional points. Indicated *p*-values are based on the Kruskal–Wallis statistical test.

We compared infants in the control cohort at three different ages with the three sequence settings to better characterize typical development of the infants' HM in these different interactive settings (Figure 4). The infants' HM were very similar at early stages of development (three to 4 months), irrespective of the descriptor (non-significant *p*-values) in the three interaction sequences. By 5–6 months, HM characterizing the interaction sequence with the cuddly giraffe were significantly different from those of the other two sequences for nine of the 16 descriptors. The largest difference was seen for descriptors related to movement acceleration (accSd, accMax; *p* < 0.001) and the surface explored (ampMax, yRange; *p* < 0.002). The differences in the infants' HM in the cuddly giraffe sequence, with respect to those of the other two sequences, further increased after the seventh month, when most of the descriptors became statistically different. At this point, the biggest difference was for surface exploration, especially vertical amplitude (yRange, *p* < 0.0001), reflecting reaching gestures (Figure 4, top panel). The difference observed in the overall time spent in movement for this sequence (Figure 4, bottom panels) became statistically significant (*p* = 0.01) only after the seventh month.

### Kinematics of infants' HM: time points and cohort

**Figure 5:**
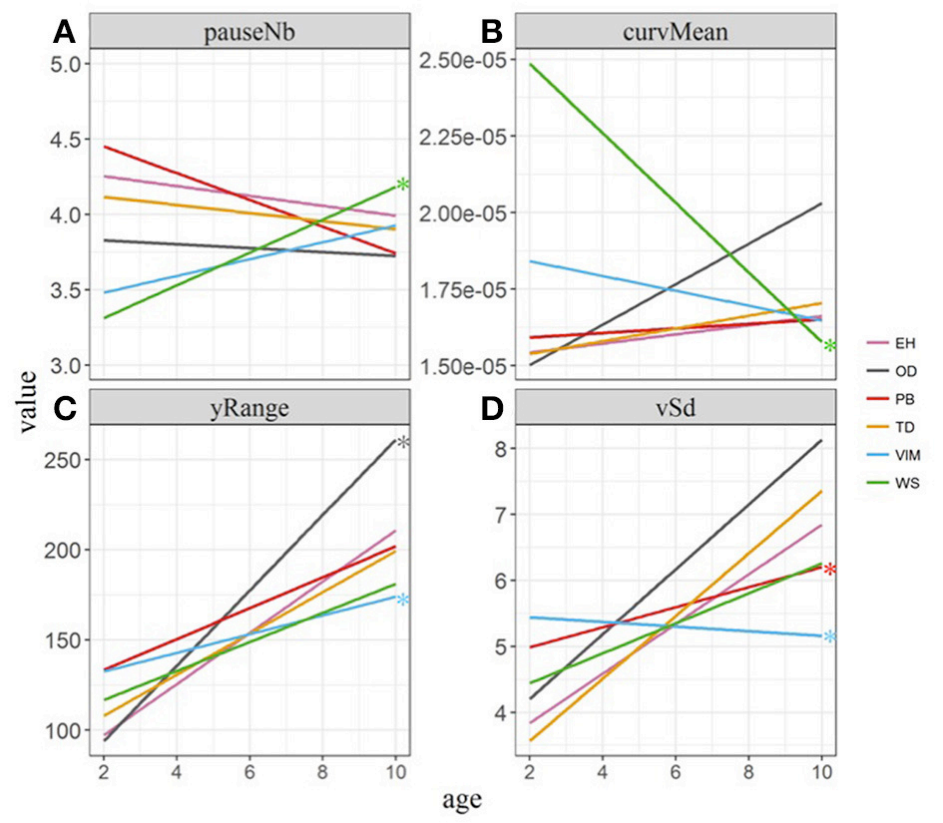
Evolution of the descriptors of hand movements with age. The color code represents the infant cohort. A linear model was built for each cohort independently. Asterisks indicate cohorts with an age evolution statistically different from that of the Control cohort. **(A)** Number of pauses: interaction age × cohort is significantly different between the WS and Control cohorts. **(B)** Average curvature: interaction age × cohort is significantly different between the WS and Control cohorts. **(C)** Range of the y coordinates: interaction age × cohort is significantly different between the OD and VIM cohorts and Control cohorts. **(D)** Velocity standard deviation: interaction age x cohort is significantly different between the PB cohort, the VIM and the control cohorts.

We estimated the interaction between age and cohort in a linear regression model to compare the developmental trajectories in the clinical cohorts, taking into account all the three sequences. Representative results of the models are shown in Figure 5. The movements of the infants with the VIM cohort evolved differently from those of the control cohort for several descriptors related to velocity, acceleration, amplitude, and extreme curvature of the HM, with an observed increase that was slower than that of the control cohort (sometimes null and even the reverse) (Figures 5C,D, blue line). In the PB cohort, the evolution of the variability of acceleration and velocity was significantly lower than that of the controls (Figure 5D, red line). The OD cohort showed a stronger increase than the controls for descriptors associated with the vertical amplitudes (ySd and yRange) of the movements (Figure 5C, gray line). The WS cohort differed significantly from the controls for the pause and curvature descriptors (pauseNb, curvMean, and curvMax), with an increase in the number of pauses (Figure 5A, green line) and a decrease in the average curvature of the movements (curvMean) between 3 and 10 months, whereas the control cohort presented no significant evolution (Figure 5B, green line) for either of these descriptors.

We performed a specific analysis of the WS cohort, using both chronological age (CA) and DA, to evaluate whether the difference in evolution of the HM in the WS cohort was associated with the infants' development. Among the 13 descriptors showing an age effect in the population of all cohorts, six (vMean, vSd, accSd, accMeanMov, decMaxMov, and ampMax) exhibited no significant evolution with CA for the WS cohort, but showed a significant evolution when the global DA was considered.

**Figure 6:**
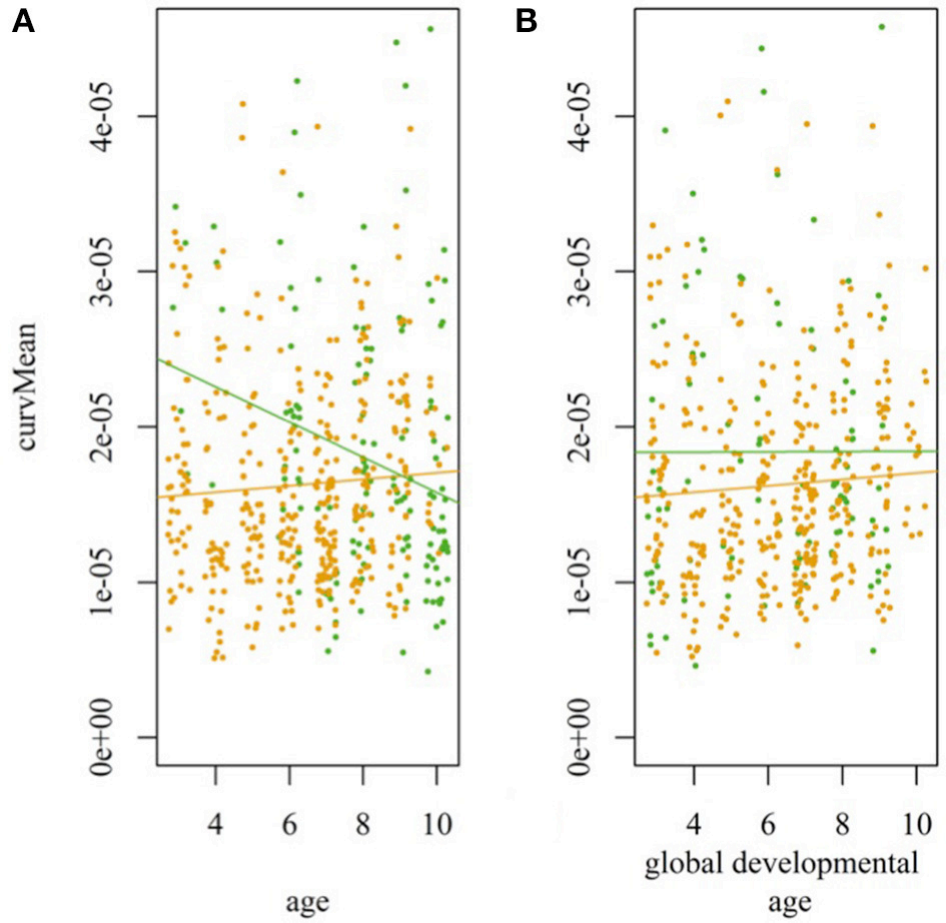
Evolution of the average curvature of the hand movements in the WS cohort. Evolution of the average curvature of the hand movements as a function of chronological age **(A)** and global developmental age **(B)** for WS (green) and control (orange) cohorts. The linear models are shown to illustrate the significant difference in evolution when the age of the infants is considered as the variable (*p* = 2e-4) and the non-significant difference when the global developmental age is considered as the variable (*p* = 0.59).

We tested whether interactions remained significant for descriptors that showed an interaction between age and cohort and differentiated the WS cohort from the control cohort (pauseNb, curvMean, and curvMax), when the DA was used instead of the CA for the WS cohort (Figure 6). There was no interaction for the number of pauses (not shown) nor average curvature (Figure 6B) when DA was used for the WS cohort relative to the control cohort, indicating that their evolution was similar. The difference in the evolution between the WS and control cohort was still present despite this age correction for the descriptor maximum attained curvature (curvMax), which reflects greater jerkiness of the movements.

## Discussion

Here, we aimed to characterize HM in dyadic interactive situations, in infants at risk for developmental disorders and controls. We used kinematics of HM in three different contexts to study 94 infants, aged 2–10 months, divided into six cohorts. We setup a technical environment to reproduce and detect the main features of HM that have been previously described to be relevant, such as speed, acceleration, and deceleration; curvature; exploration of space; and pauses (von Hofsten, 1991; Thelen et al., 1993, 1996; Berthier and Keen, 2006; Meinecke et al., 2006). Not surprisingly, our results confirm the strong correlation of descriptors of HM representing the same domain (speed, curvature, explored surface, and interruptions). We also observed a strong correlation between curvature and speed (both the mean velocity and mean acceleration) of the HM. This correlation is well-known and has been associated with the functioning of the central control mechanisms in typical infant development (Viviani and Schneider, 1991; de'Sperati and Viviani, 1997).

Our results support the relevance and reliability of kinematic features of HM in a natural interactive face-to-face setting. Furthermore, they provide the first longitudinal study using kinematic analysis of HM in a natural and replicable interactive face-to-face context between infant and mother, in TD infants and several cohorts at risk for developmental disorders. Previous studies have analyzed spontaneous infant movements, but without any interactive context.

We showed a significant association of the kinematic features of HM with age in all cohorts. HM become more complex, with larger and modulated movements, which were gradually used for more sophisticated goals. This is consistent with previous studies that have reported increased hand velocity with age in reaching (Halverson, 1933). Fallang et al. (2000) also reported an increase in the length of the displacement-path from 4 to 6 months of age. Konczak et al. (1995) showed an increase in trajectory length in babies from 4 to 15 months of age, and an increase in Vmax, which was not statistically significant. However, other studies did not find a developmental influence on these variables in reaching (Mathew and Cook, 1990; von Hofsten, 1991) and none were performed during interactive sessions.

We also showed that HM differ depending on the experimental interactive context (interacting with an object or a person). We longitudinally studied HM in the TD cohort in three types of sequences: with a “cuddly giraffe,” an object-directed setting (reaching, exploring); and two other interactive settings (free play and nursery rhyme). None of the HM descriptors revealed differences between the free play, cuddly giraffe, and the nursery rhyme sequences at 3–4 months of age, showing that object-directed and interactive-directed movements did not differ at this developmental stage. At 7–8 months, however, the HM showed statistically significant differences between the cuddly giraffe vs. the other two scenarios. The infants' HM showed larger amplitudes, higher velocity and accelerations, and more overall time spent in movement in the cuddly giraffe episode.

This is the first study to investigate the evolution of HM into gestures, during the transitional period from the beginning of goal-directed movements to interactive-directed gestures, in a prospective and interactive manner. Our findings support the assumption that movements show a shift in their characteristics at 5–6 months of age, depending on the target (Zoia et al., 2013). Similar toy-oriented changes during early arm movements (around 3½−4 months of age) have been described using kinematics in the pre-reaching period (Bhat and Galloway, 2006). This shift probably corresponds to a period when communicative gestures begin to individualize. Typically developing infants begin to intentionally communicate with their caregivers at ~7–9 months of age (Crais et al., 2004; Iverson and Goldin-Meadow, 2005; Guidetti and Nicoladis, 2008). The quality of this communication is a good predictor of early joint attention, an information-processing system that begins to develop at ~4–6 months (Mundy et al., 2009).

Our findings strongly support very early infant competence for communicative skills. The transition from HM to communicative gestures, at the beginning of the second semester of life, is a transactional process. This implies bidirectional influences through continuous and dynamic interaction cascades between infants, their neural equipment, and their environment, in epigenetic and probabilistic models (Sameroff and MacKenzie, 2003; Gottlieb, 2007; Masten and Cicchetti, 2010; D'Souza and Karmiloff-Smith, 2017).

We also showed that specific environmental and developmental factors shape the developmental trajectories of HM in different cohorts at risk: environmental for VIM, developmental age for WS and PB, and both for OD. Our data show that a specific environment (blind mother, orality disorders) shapes HM pattern constructions from very early on. Infants with VIM showed a different pattern of HM (acceleration, speed, jerk and vertical amplitude), characterized by relative stability from 2 to 10 months. Two possible explanations can be suggested. First, the movements of VIM infants may be influenced by the different maternal gestures, from very early in their development. This interpretation is in agreement with Lederman and Klatzky (1987), who showed that the tactile and kinesthetic exploratory procedures of blindfolded subjects differ from those of controls, leading to a sequential process of exploration. It is also possible that infants with VIM notice that their mother is not able to see their movements, so they spontaneously do not increase the complexity of their HM. The fact that infants with OD show larger vertical amplitudes in their movements than the other cohorts could be due to the particular importance of the mouth and orality in this cohort. Their disease is characterized by special feeding procedures (special diet or nasogastric feeding tube). It is possible that babies with tube feeding direct their hands more often to their faces to touch the nasogastric tube and/or their mouth. An early oral stimulation program accelerates the transition to full oral feedings in preterm infants (Fucile et al., 2002). We can therefore hypothesize that this infant's oral self-stimulation could be an adaptive behavior. We did not find any significant characteristic for EH infants, possibly due to the small size of this cohort.

Finally, our results provide strong evidence that the kinematics of HM have specific patterns in the cohort with neurodevelopmental disorders. A core deficit was found in infants with WS. The curvature of HM, or jerkiness, showed no influence of age in WS relative to the other infants when we considered the DA. However, the absence of an influence of age on average speed and its variability, average acceleration and its variability, maximum deceleration, and the maximum movement amplitude relative to the other infants, disappeared if we considered the DA of infants in the WS cohort, and thus appears to be associated with intellectual disability. Torres et al. (2016) detected very early stunting in the development of voluntary neuromotor control in newborns tracked longitudinally for 5 months. The fluctuations of motor performances, especially the transition from spontaneous random noise to a systematic signal, allowed them to detect specific features in at-risk infants for neurodevelopmental disorders. Here, the jerkiness (assessed by the descriptor maximum attained curvature, decreased curvMax), which clinically translates to smaller movements in WS infants, is the only parameter that remained significantly different in the WS cohort, even after correction for DA, and is probably a specific feature of this cohort at high risk for autism. This is of importance, because no specific core deficit in clinically assessed movement at early stages of development has been found in infants at high risk for autism (Adrien et al., 1993; Zwaigenbaum et al., 2005 for prospective ones, 2013; Ozonoff et al., 2008 for retrospective studies, Landa and Garrett-Mayer, 2006). Only Teitelbaum et al. (1998) found asymmetric and unusual movements, along with delayed maturity of movements. Bhat et al. (2012) showed that motor delay at 6 months was predictive of a communication delay at 18 months for the high-risk group, but was not specific to autistic symptoms. The difference in jerkiness could be the first specific sign of later more repetitive hand motor patterns, such as flapping. Philippi et al. (2014) showed that kinematics (the lack of variation in HM in 3-month-old premature babies, interpreted as stereotypical movements) was the best predictor of future cerebral palsy. Goodwin et al. (2011) noted that kinematics identified repetitive patterns better than clinicians in assessing a video of autistic infants. Kinematics could provide earlier and more specific information than clinicians on developmental problems in general, and those specific to ASD. Further investigation is needed, depending on the evolution of these babies, to confirm this assumption, as the cited studies were not assessed during specific interactive sessions.

Our findings of a slower developmental trajectory in the acceleration and velocity of movements in PB infants are consistent with previous reports, suggesting that infants born preterm demonstrate impaired object exploration behaviors throughout infancy and toddlerhood (Lobo et al., 2015). In this case, the poor acceleration and velocity of movements found in the present study, could predict some aspects of developmental outcome from very early on, despite the absence of a neurological disorder. Several authors have predicted later neurodevelopmental outcomes of infants by the single preterm assessment of GM. Here, our findings indicate a similar tendency, but found in later stage movements. However, the environment has also been shown to positively affect development in preterm babies. In two different experiments, Peña et al. (2014) demonstrated that 7-month-old preterm infants performed as well as 7-month-old full-term infants (with whom they shared the same CA) and not like 4-month-old full-term infants (with whom they shared the same postmenstrual age). They conclude that the duration of exposure to visual experience thus appears to have a greater impact on the development of early gaze following than does postmenstrual age, showing that early exposure to face-to-face interactions with other humans helps preterm babies acquire this capacity sooner than full-term infants of the same CA, despite their immature brains.

## Study limitations

This study has some limitations. We tracked information only on the movement of the right hand. This was however also the case in other studies (Philippi et al., 2014, in 3-month-old infants with cerebral palsy).

## Conclusions and future work

This study highlights the relevance of automated assessment, such as the kinematics, of HM in infants at risk, in an interactive and longitudinal setting, in the early assessment of developmental anomalies in communicative skills that were difficult to find by clinical examination. Kinematics will become easier to set up in natural contexts in the near future, because methods like RGB-D sensors (e.g., Kinect) allow online extraction of body movement cues and their temporal evolution within interactive ecological contexts (Avril et al., 2014). Kinematics are particularly well-suited to capture important developmental processes during the period in which movement develops from object- to person- directed gestures, and has been shown to detect early clinical signs of possible later disorders earlier and more accurately than clinicians. This setting could be used to closely follow infants at risk of developmental disorders during their first year of life to better identify specific targets for treatment. Our findings provide strong evidence for early preverbal communicative skills. This period is influenced by biological and environmental factors. Both appear to play a role very early in the developmental process: intrinsic movement features are modified in preterm birth and West syndrome cohorts, and the environment affects the movements of infants of VIMs. We will continue to study the developmental trajectory in these different cohorts to assess whether these influences affect later skills, such as joint attention, social cognition, and spoken language. Studies of longitudinal cohorts are of great interest to explore developmental and experience factors (Thomas et al., 2009; Spittle et al., 2016). Our cohorts already constitute a large and diversified clinical database that will provide information concerning the complexity of development, particularly in high-risk infants, which should help early prevention and rehabilitation. Future work may improve technical aspects of kinematics, include novel cohorts of at risk infants such as deaf infants (Beers et al., 2014), integrate these preliminary data by establishing large research cooperation at multiple levels (e.g., genetic, epigenetic, neuronal, cognitive, environmental, social).

## Author contributions

LO, M-TL, BG: Conception; LO, M-TL, MA, BG, MG-K: Design; KB, XJ, XC: Technical conception and realization; LO, ML, CG, RS, JW, ET: Clinical work; ST, TDS, MG-K: Statistical work. LO, M-TL, MA, MG-K: Paper writing.

### Conflict of interest statement

The authors declare that the research was conducted in the absence of any commercial or financial relationships that could be construed as a potential conflict of interest. The reviewer, DC, declared a past co-authorship and a shared affiliation with one of the authors, KB, to the handling Editor.

## Abbreviations

ASD: Autism spectrum disorders
CA: chronological age
DA: developmental age
EH: early hospitalization
GM: general movements
HM: hand movements
OD: orality disorders
PB: preterm birth
TD: typical development
VIM: visually impaired mothers
WS: West syndrome.
