# Supplementary material for: Developmental Trajectories of Hand Movements in Typical Infants and Those at Risk of Developmental Disorders: An Observational Study of Kinematics during the First Year of Life
Source: Front Psychol. 2018 Feb 19;9:83. doi: 10.3389/fpsyg.2018.00083 (PMC5826068; doi:10.3389/fpsyg.2018.00083)
Supplement: Supplementary file 1 [file Presentation1.zip › software/HowToExtractHandTrajectories.pdf]

How to extract Hand Trajectories

(Ref: Lisa Ouss et al., *Developmental trajectories of Hand Movement in infants at risk: a follow up study during the first year of live*, submitted to Frontiers in Psychology, 2017)

Warning: the first software named “extraction_main.exe” has been developed under Windows XP (SP2, 32 bits version); operation under newer OS is not guaranteed. The second step of processing uses a custom script in Matlab.

1. Copy the directory named “extraction” to a chosen location; for example, your Desktop.

2. Put a sequence in the subdirectory “sequences” (there is already a sequence named “test.avi”)

3. Run the program "extraction_main.exe" by double clicking on it.

4. A console window appears:

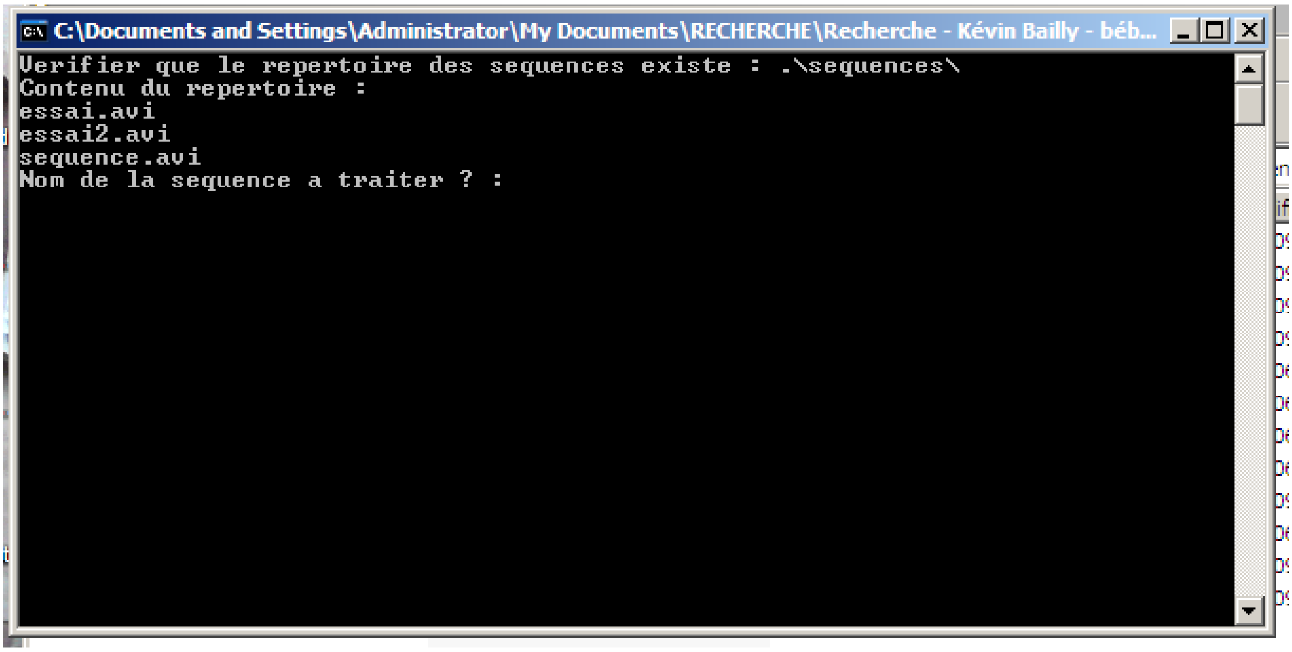

This window displays the names of avi sequences contained in the subdirectory “sequences” (here, “essai.avi”, “essai2.avi” and “sequence.avi”).

1. Write the filename of the sequence to process, and push the “enter” key.

2. Choose the hand (right hand or left hand) to process; write “0” for the right hand (= “main droite” in french) or “1” for the “left hand” (= 'main gauche” in french) then push « enter » key.

3. a second window with scrollbar (up) and the first image of the chosen sequence (bottom) appears:

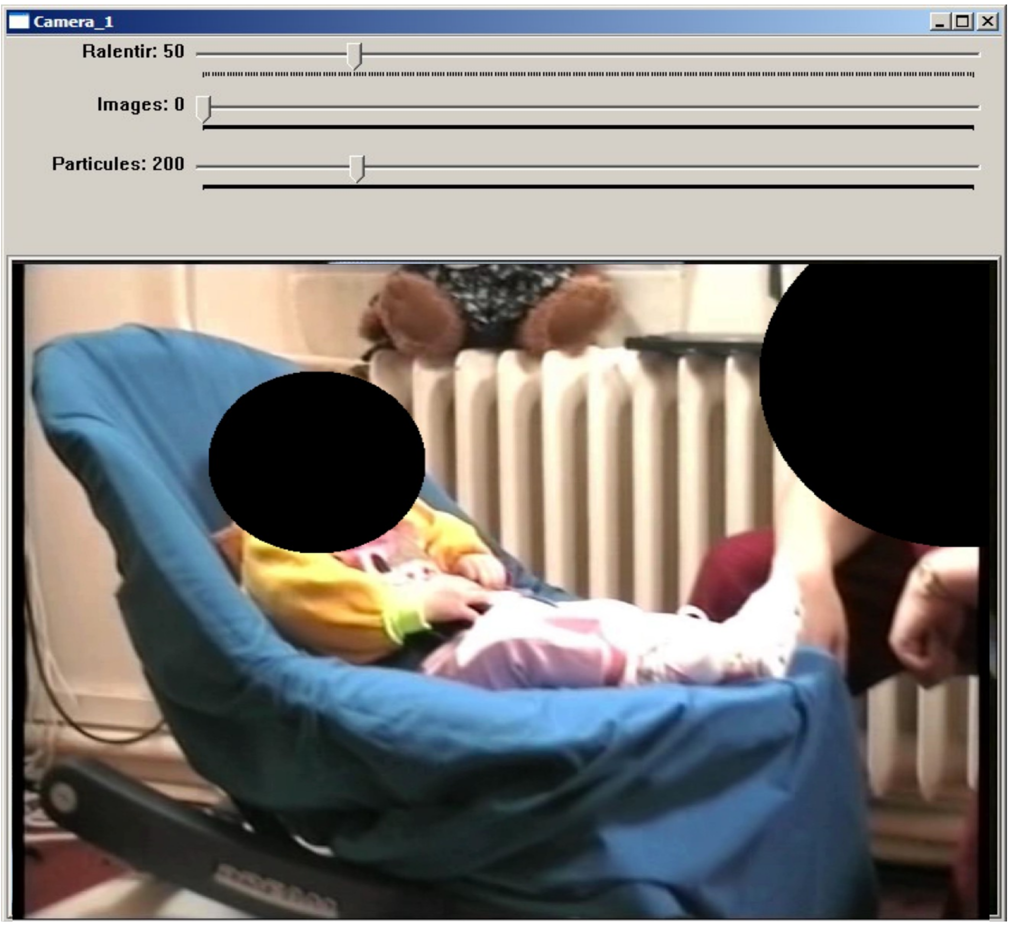

4. With the mouse, move the scrollbar named “Images” in order to choose an image wherein the armhand corresponding to the chosen hand (green armband=right hand; red armband=left hand) is fully visible.

5. Move the mouse pointer (a cross) to the armband center; then right click.

6. Wait for a second and then click on the console window and press any key.

7. The program will automatically track the armband in the further images. To accelerate it, we can put the scrollbar named “Ralentir” to 0.

8. When the program has processed the last images, click in the console window and push any key to quit it.

The locations of the hand are automatically recorded in a text file saved in the directory “sequences”. The filename will be the concatenation of the sequence's name and an indication (in french) of the chosen hand (“sequence.avi” + “right hand” → « sequence.avi_main_droite.txt »).

First column of the recorded data concerns the number of the image, the second the X-coordinates and the third the Y-coordinates.

The data may be incomplete if the tracking partially failed. This can due to:

- an occlusion of the armband,
- a high-speed movement of the hand (the image is then blurred).

This could be due to a user's bad choice of the image or to a miss click; in this case, repeat instructions 1 → 8.

A script running under Matlab and named « complete_sequence.m » (in directory named “extraction”) will allow you to manually complete the data.

9. Run « Matlab ».

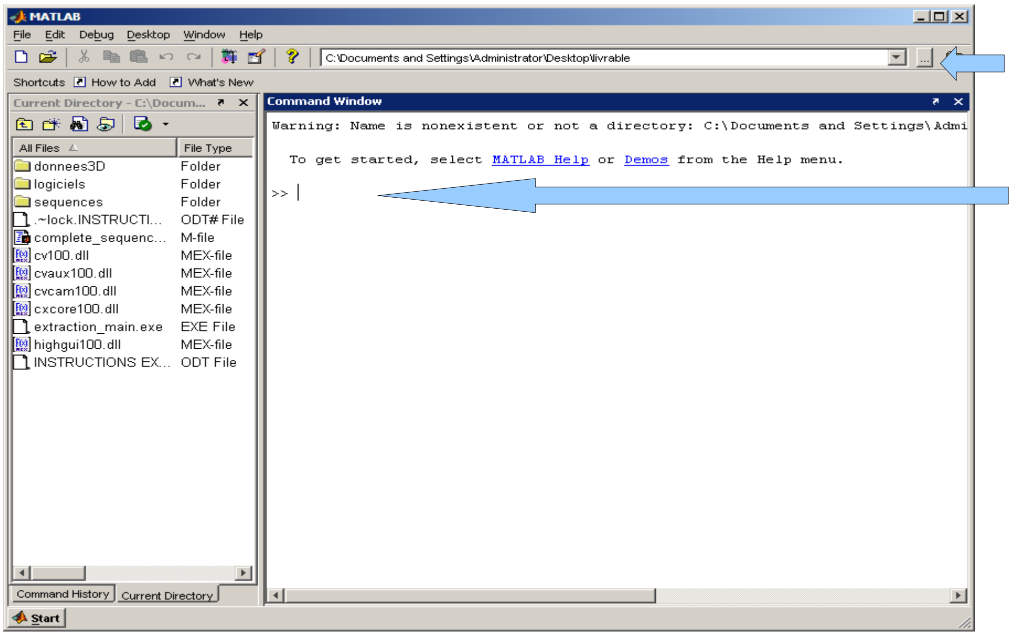

“...”

Command window

10. Click on “...” and select the directory “sequences”.

11. Write in “complete_sequence” in the command window, and press “enter” key.

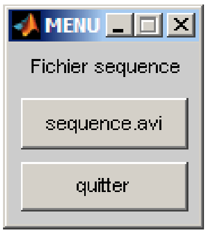

12. A menu window appears. Click on the name of the sequence to complete (example : sequence.avi”).

13. A new menu appears. Click on the text name of the recording trajectory to complete (Example : “sequence.avi_main_droite.txt”).

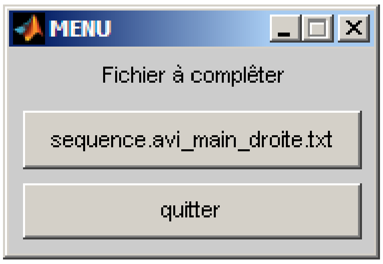

14. A new window appears; it will show the images (“missed images”) that could not be correctly processed by the “extraction” program.

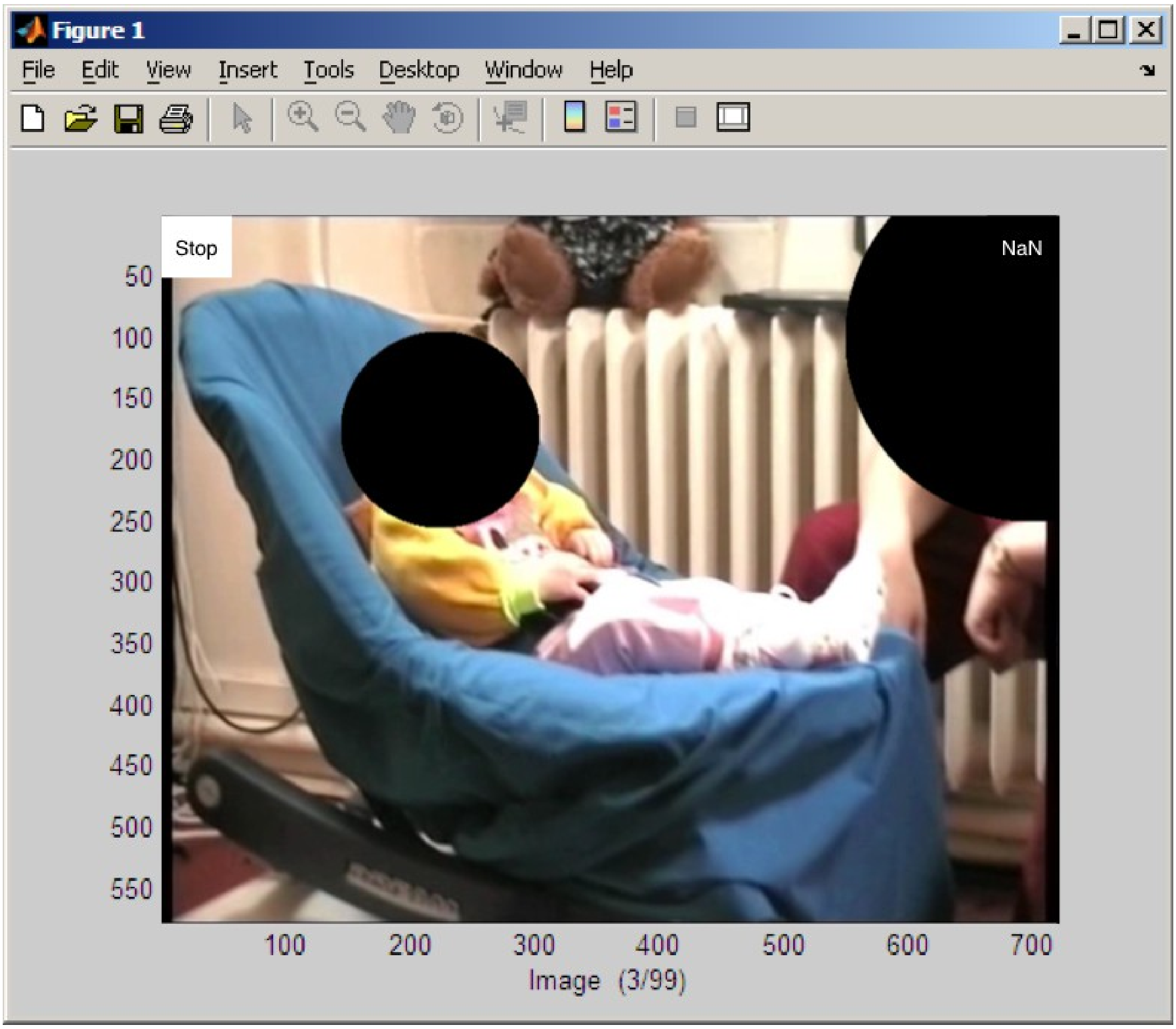

15. Move the mouse pointer to armband and right click.

16. The next missed image will be shown.

17. Continue instructions 15 and 16 until you reach the end of the sequence. A counter (bottom) indicates the number of manually processed images and the number of the images to process.

Remarks :

- Before processing text files, it is advisable to copy them to another directory (in order to avoid any wrong operation that could erase them).
- You can stop at any moment by clicking on the upper-left white part of the image (named "stop"); the program will record your work in the text file. You can complete it later.
- If you cannot see the armband, you can click on the upper-right black part of the image (named "NaN"). The program will then show the next image to process. (In the text file, the line corresponding to this non-processable image will contain the values "-1" instead of the coordinates).
